# Effect of physiotherapy rehabilitation on stage 4 avascular necrosis of femur following pyogenic arthritis: a case report

**DOI:** 10.11604/pamj.2022.41.17.32883

**Published:** 2022-01-07

**Authors:** Akshaya Virendra Saklecha, Om Chandrakant Wadhokar, Deepali Swapnil Patil, Waqar Mohsin Naqvi

**Affiliations:** 1 Department of Musculoskeletal Physiotherapy, Ravi Nair Physiotherapy College, Datta Meghe Institute of Medical Sciences (DU), Sawangi Meghe, Wardha, Maharashtra, India,; 2 NKP Salve Institute of Medical Sciences, Nagpur, Maharashtra, India

**Keywords:** Avascular necrosis, femur, pyogenic arthritis, limb length discrepancy, physical therapy, case report

## Abstract

Avascular necrosis of the femur is a painful condition marked by a disruption in the blood supply to the femoral head, which causes the femur bone to distort; characterized by pain and restriction of movements at the affected joint with a limp. The study aimed to provide a case of nontraumatic stage-4 avascular necrosis of the left femoral head with gross 40-degree adductor deformity. In this report, a 27-year-old female complained of pain in her left hip joint and difficulty in walking. She was a known case of pyogenic arthritis with 5 cm of true shortening on her left side and a gross 40-degree adduction deformity of her left leg. According to Ficat and Arlet's grading system, an X-ray showed stage-4 avascular necrosis of the left femoral head. For this, she was managed with adductor tenotomy, medications, and physiotherapy management with a one-month rehabilitation protocol. A physiotherapy intervention consists of a non-weight-bearing phase and a weight-bearing phase. At the time of her physiotherapy discharge, the patient experienced alleviation from symptoms and achieved functional mobility that she had previously been unable to tolerate owing to pain. As a result, physical therapy rehabilitation has been proved to be highly beneficial. This case study concludes that multidisciplinary team including medical, surgical approach and physiotherapy rehabilitation played a vital role in reducing pain; enhance muscle strength, functional independence, and quality of life in patients with stage 4 avascular necrosis of the femur followed by pyogenic arthritis.

## Introduction

Avascular necrosis of the femur is also known as osteonecrosis [1]. It is a painful disorder marked by disruption in the blood supply to the femoral head, which causes the femur bone to distort [2]. The head of the femur has a very limited blood supply, with only a few anastomoses leading to a wedge-shaped region of avascular necrosis (AVN) [3]. Thus, the head of the femur is the most often impacted site [4]. It usually leads to irreparable joint degeneration, resulting in considerable disability as a result of pain and movement restrictions. The prevalence rate of avascular necrosis was found to be 8% with an age range of 27 years at the time of AVN diagnosis ranging from 18 to 54 years of age [5]. The aetiology of AVN may be posttraumatic, nontraumatic or idiopathic. It was characterized by pain, which is felt mostly in front of the joint and restriction of movements at the affected joint with a limp. Pathophysiology covers a number of things. Cell death follows a predictable pattern as a result of a compromised terminal artery supply to the bone and a complex process of bone resorption and formation [6]. During the necrosis phase, the joint cartilage that covers the femoral head is damaged, this accelerates the wearing. The joint gradually deforms the femoral head, its height decreases and causing the lower limb to shorten [7]. The Ficat and Arlet classification [8] combines a mix of radiography, magnetic resonance imaging, and clinical features to radiologically stage avascular necrosis [9, 10]. Avascular necrosis (AVN) medical management is primarily determined by the location and severity of the disease [11]. Medical management includes Pharmacological Therapy, core decompression, osteotomy and total hip arthoplasty. Physical therapy plays an important role in this condition by reducing the symptoms and enhancing the functional independence and quality of life. This report provides a case of a female complained of pain in her left hip joint and difficulty in walking due to adductor deformity of the left leg with a limb length discrepancy and diagnosed with stage-4 avascular necrosis of the left femoral head.

## Patient and observation

**Patient information:** the patient in this case is a 27-year-old woman from Gondia who has been complaining of pain in her left hip for the past 10 years and has had difficulty walking on occasionally for the past 15 years. A few years back, the patient was apparently alright; then, she gradually started pain over the left hip. The pain is insidious in onset and gradually progressive in nature with an intensity of 8/10 on nural pain rating scale (NPRS). It is dull aching in nature which gets aggravated by movements and exertion and relieved by rest and medication. The pain progressed over five years to the extent to which the patient had difficulty in day-to-day activities like sitting, cross-legged, and squatting. She had a previous history of infection at multiple sites of the body, after which her left lower limb became short, and she started feeling occasional pain over the hip; for that, she visited the local physician in gondia where investigations were carried out that revealed pyogenic arthritis. For that, she had taken the medicines. There was some relief of pain, but a few days later, she had the same complaints, so she decided to visit the Orthopedic Department of AVBRH in November 2021 with a complaint of pain over the left hip and difficulty in walking. An X-ray was carried out as advised by the consulted orthopedic surgeon. X-ray of her pelvis showed over ridding of the femur, and there was the destruction of femoral head and neck and left-sided tilt of the pelvis. She was diagnosed with stage-4 avascular necrosis of the left femoral head with gross 40-degree adductor deformity of the left limb and admitted to the orthopedic department, ward unit III, for further management. Forty (40)-degree adductor deformity was managed by adductor tenotomy on November 23^rd^, 2021. On November27^th^, 2021 patient started Physiotherapy rehabilitation with the proper protocol.

**Figure 1:**
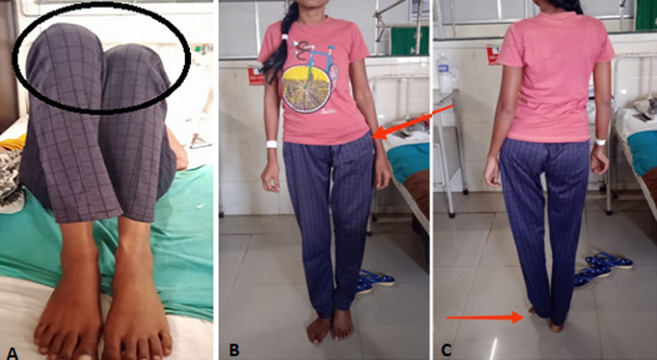
(A,B,C) pre-rehabilitation figure shows the limb length discrepancy of the left leg

**Figure 2:**
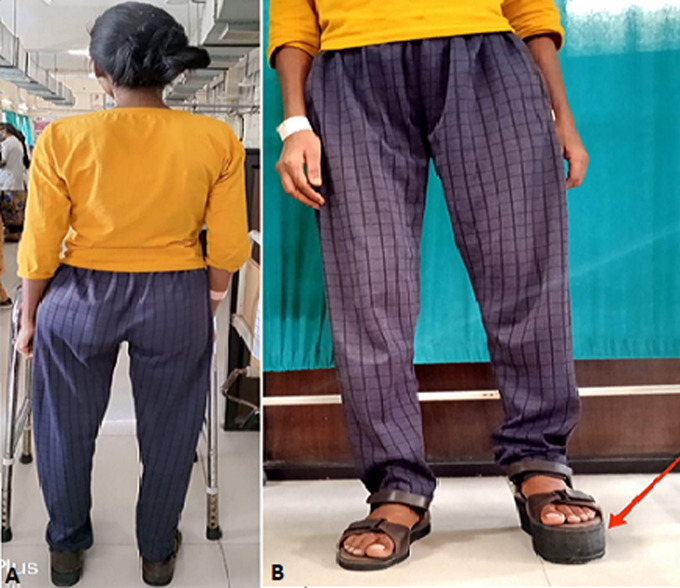
(A,B) post-rehabilitation figure; arrow represent the patient wears the shoe raised of 2 inches

**Clinical findings:** the patient was assessed with the left ASIS at a higher level in a supine position. On general examination, the patient's vital signs were normal: afebrile fever, PR 86 beats/min, respiratory rate 18 breaths/min, Blood pressureP 126/80mmHg, and BMI 26.04 kg/m^2^. The underlying skin appeared to be normal upon examination. The patient maintained 15-degree adduction on the left lower limb, which looks to be about 5cm shorter than the right lower leg. A gross adduction deformity of roughly 40 degrees was found at the hip joint, and scoliosis and pelvic hiking of left side were noticed during a posture evaluation. The local temperature was not increased on examination of the left hip; grade-2 tenderness was present over the left anterior joint line. There was 5cm of true shortening and 7cm of apparent shortening of the left leg (Figure 1). The left (affected side) hip's range of motion was substantially limited and painful in all ranges (Table 1). The range of motion of the lumbar spine was normal, although there was pain in lumbar flexion and lateral flexion. The muscle of the left thigh likewise showed signs of atrophy. The abductor's muscle and the hamstring muscle of the affected side were tight; the ober test was positive. Normal reflexes and sensory testing were found during the neurological examination on both sides. Muscle weakness was seen in the left lower limb; in comparison to the right lower leg (Table 2). The distal circulation was intact. The gait pattern was altered. During walking patient wears the shoe raised of 2 inches to compensate for the limb length discrepancy (Figure 2).

**Table 1:** pre-rehabilitation range of motion

Joint	Right active	Right passive	Left active	Left passive
**Hip**				
Flexion	0-110°	0-120°	0-60	0-90
Extension	0-105°	0-110°	Unable to perform	0-20
Abduction	0-35°	0-45°	Unable to perform	0-40
Adduction	30-0°	45-0°	0-15	0-40
Medial rotation	0-40°	0-45°	0-30	0-5
Lateral rotation	0-40°	0-45°	Unable to perform	0-40
**Knee**				
Flexion	0-125°	0-130°	0-110	0-130
Extension	125-0°	130-0°	110-0°	130-0
**Ankle**				
Plantarflexion	0-50°	0-50°	0-45°	0-50°
Dorsiflexion	0-20°	0-20°	0-18°	0-20°
Inversion	0-35°	0-35°	0-27°	0-35°
Eversion	0-25°	0-25°	0-25°	0-25°

**Table 2:** pre muscle strength testing

Muscles	Right	Left
Hip		
Flexors	4/5	3/5
Extensors	4/5	3/5
Abductors	4/5	3/5
Adductors	4/5	3/5
Knee		
Flexors	5/5	5/5
Extensors	5/5	5/5
Ankle		
Plantarflexors	5/5	4/5
Dorsiflexors	5/5	4/5
Invertors	5/5	4/5
Evertors	5/5	4/5

**Timeline of current episode:** on March 2006: she was diagnosed with pyogenic arthritis. September 2020: the patient had limb length discrepancy of left leg. November ^th^, 2021: the patient was admitted to the orthopedic ward. November 12^th^, 2021: X-ray was performed and diagnosed with avascular necrosis of the left femoral head with gross 40-degree adductor deformity of the left leg. November23^rd^, 2021: adductor tenotomy was conducted. November 27^th^2021: physiotherapy rehabilitation was started.

**Figure 3:**
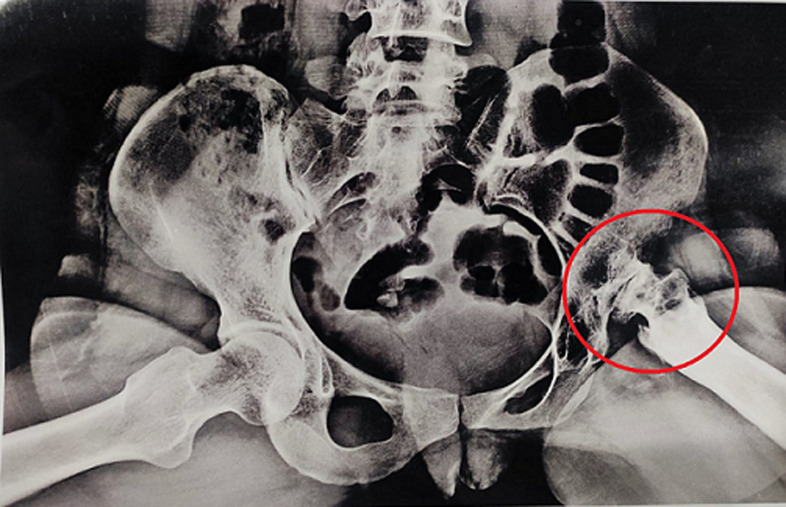
X-ray of pelvic showing grade IV avascular necrosis of left femoral head

**Diagnostic assessment:** red blood cell (RBS), complete blood count (CBC), liver function test (LFT), kidney renal rest (KFT) were carried out. In any of these examinations, no changes were found. The diagnostic tool performed was an X-ray. Due to the family's financial insecurity, computed tomography (CT) scans and magnetic resonance imagin (MRIs) were not performed. An X-ray of the pelvis revealed a left-sided tilt of the pelvis. The femoral head and neck are damaged, with the remnant abutting on the outer part of the acetabulum Ilium, resulting in a false joint. The lesser trochanter is just about 4-5cm long. The original acetabulum is significantly undeveloped, shallow, and in the process of forming new bone. The proximal third femur is sclerotic, with an uneven and narrow medullary canal with lysis in the trochanteric region. Her imaging at this point indicated grade IV avascular necrosis of left femoral head (Figure 3).

**Diagnosis:** the findings are in accordance with avascular necrosis of head of femur. A diagnosis of stage 4 avascular necrosis of the left femoral head with gross 40-degree adductor deformity was made.

**Therapeutic interventions:** physiotherapy intervention was initiated and consists of two phases: non-weight-bearing phase and weight-bearing phase (Table 3).

**Table 3:** physiotherapy treatment protocol

Exercise protocol	Duration and frequency	Rationale
	**Phase A: non-weight-bearing phase**	
Ankle toe movements	20 repetitions for three times a day	To prevent pedal oedema
Isometrics to quadriceps, hamstrings and glutei	10 repetitions with 5 seconds hold for 2 times the day	To minimize muscle atrophy and maintain contractility of the muscle
Strengthening exercises to hip, knee and ankle	10 repetitions with 5 seconds hold for 2 times the day	To improve muscle strength and endurance
ROM exercises in supine and standing	10 repetitions 2 times the day	To regain and maintain range of joints
	**Phase B: weight-bearing phase**	
Ambulation with walker	For 10 minutes thrice a day	To improve patients lung compliace

**Follow-up and outcome of interventions:** on (Table 4).

**Table 4:** pre and post rehabilitation range of motion

Pre treatment			Post treatment	
Joint	Active	Passive	Active	Passive
**Hip**				
Flexion	0-60	0-90	0-90	0-110
Extension	Unable to perform	0-20		0
Abduction	Unable to perform	0-40	0-35	0-40
Adduction	0-15	0-40	0-35	0-35
Medial rotation	0-30	0-5	0-30	0-30
Lateral rotation	Unable to perform	0-40	0-25	0-35
**Knee**				
Flexion	0-110	0-130	0-125	0-130
Extension	110-0°	130-0	125-0	130-0
**Ankle**				
Plantarflexion	0-45°	0-50°	0-50°	0-50°
Dorsiflexion	0-18°	0-20°	0-20°	0-20°
Inversion	0-27°	0-35°	0-35°	0-35°
Eversion	0-25°	0-25°	0-25°	0-25°

**Patient perspective:** “I had the physiotherapy treatment, which has helped me heal faster, walk independently, and improve my day-to-day activities, which I was unable to accomplish before the physiotherapy treatment.”

**Informed consent:** the patient was first informed about the study, and then informed consent was obtained.

## Discussion

Avascular necrosis is a degenerative bone disease marked by the loss of bone cellular components due to a disruption in the subchondral blood supply. Osteonecrosis, aseptic necrosis, and ischemic bone necrosis are all terms used to describe this condition. At weight-bearing joints, it usually affects the epiphysis of long bones [1]. The head of the femur has a very limited blood supply, with only a few anastomoses leading to a wedge-shaped region of avascular necrosis (AVN) [3]. Thus, the head of the femur is the most often impacted site [4]. It usually leads to irreparable joint degeneration, resulting in considerable disability as a result of pain and movement restrictions [12] (Table 5). In this case, the patient presented with avascular necrosis of left femoral head with deformity following pyogenic arthritis. The possible cause in this case was pyogenic arthritis. Physiotherapy rehabilitation helps to reduce pain, improves strength and quality of life. As in the study by Manal K Yousef 2014, effect of physical therapy on range of motion and pain in cases of femoral head avascular Necrosis [13]. Physical therapy for femoral head osteonecrosis was compared in 38 persons with sickle cell disease by Marchese *et al*. for the first 6 weeks of the trial, all patients were subjected to toe-touch weight-bearing limitation on the afflicted hip as part of the physical therapy protocol.

**Table 5:** pre and post rehabilitation muscle strength

Muscle strength testing		
Muscles	Pre-treatment	Post-treatment
**Hip**		
Flexors	3/5	4/5
Extensors	3/5	4/5
Abductors	3/5	4/5
Adductors	3/5	4/5
**Knee**		
Flexors	5/5	5/5
Extensors	5/5	5/5
**Ankle**		
Plantarflexors	4/5	5/5
Dorsiflexors	4/5	5/5
Invertors	4/5	5/5
Evertors	4/5	5/5

Physical therapy alone looked to be equally helpful as hip core decompression followed by Physical therapy in increasing hip function, according to that study [14]. According to study done by Rahim Karim [15], osteonecrosis of the femoral head reported that exercise program was useful in enhancing the rate of patients improvement after avascular necrosis (Table 6). The present study found that, after one month of treatment, there are increase in the range of motion of the affected joints. Visual analogue scale decreased from 8 to 4/10. There was significant improvement in the strength of the muscles [16] and the patient experienced alleviation from symptoms and achieved functional mobility that she had previously been unable to tolerate owing to pain. As a result, physical therapy rehabilitation has been proved to be highly beneficial. This case study differ from others case as in this case there was involvement of multidisciplinary team including medical, surgical approach and physiotherapy rehabilitation, which played a vital role in reducing pain; enhance muscle strength, functional independence, and quality of life in patients with stage 4 avascular necrosis of the femur followed by pyogenic arthritis.

**Table 6:** pre and post rehabilitation pain rating and limb length measurement

	Pre treatment	post treatment
Pain rating (NPRS)	8/10	4/10
Limb length measurement	5cm true shortening	3 cm true shortening

## Conclusion

This case suggests that multidisciplinary approach including medical, surgical treatment and physiotherapy rehabilitation been proved to be extremely beneficial for reducing pain and improving functional independence and quality of life in patient with stage 4 avascular necrosis of the femur followed by pyogenic arthritis.
